# Caring ability of nursing students pre- and post-internship: a longitudinal study

**DOI:** 10.1186/s12912-022-00921-2

**Published:** 2022-05-30

**Authors:** Shuang Hu, Jia Chen, Renzhou Jiang, Huiping Hu, Zhonghao Hu, Xiong Gao, Wenjun Chen

**Affiliations:** 1 School of Nursing, Changsha Medical University, Changsha, Hunan China; 2 Xiangya Nursing School, Central South University, Changsha, Hunan China; 3 Yongzhou Vocational Technical College, Yongzhou, Hunan China; 4 Hunan Cancer Hospital, Changsha, Hunan China; 5 Taojiang County People’s Hospital, Yiyang, Hunan China; 6 School of Nursing, University of Ottawa, 451 Smyth Road, Ottawa, ON Canada; 7 Center for Research On Health and Nursing, University of Ottawa, 451 Smyth Road, Ottawa, ON Canada

**Keywords:** Caring ability, Internship, Longitudinal study, Nursing, Undergraduate students

## Abstract

**Background:**

Nursing students’ internship experiences may significantly impact their caring ability. However, there is a lack of comprehensive evaluation of undergraduate nursing students' caring ability pre-and post-internship in China. This study aimed to explore the differences in the caring ability of undergraduate nursing students before and after internship.

**Methods:**

The sample comprised 305 undergraduate nursing students who had undergone internships during 2018–2020 in three hospitals in Changsha, China. Caring Ability Inventory was used to measure and compare nursing students' caring ability before and after internship. Descriptive statistics and paired t-test were employed to analyze data in SPSS software (version 22.0).

**Results:**

A total of 300 students completed the survey (response rate = 98.37%). The overall score of caring ability and scores of cognitive and patience dimensions were higher after internship than before internship (*P* < 0.05). There was no significant improvement in the courage dimension (*P* > 0.05).

**Conclusions:**

Caring ability of undergraduate nursing students in China was at a low level, their overall caring ability significantly improved after the internship, indicating a positive relationship between internship and caring ability. Nursing educators and clinical nurses should emphasize the importance of caring ability development in internship planning and encourage nursing students to engage more with patients.

**Supplementary information:**

The online version contains supplementary material available at 10.1186/s12912-022-00921-2.

## Introduction

High-Quality Nursing Service project (2010) carried out by the Ministry of Health of China mention care as an integral part of clinical practice [1]. Moreover, nurses are required to provide spiritual and psychological care for patients along with excellent nursing skills [2]. Caring is the essence of professional nursing, grounded in a set of universal humanistic and altruistic values [3]. Humanistic values include kindness, empathy, concern, and love for the self and others. Altruistic values arise from commitments to and satisfaction experienced through giving others [2, 3]. Humanistic caring is a fundamental belief in the internal power of care process to produce growth and change in individuals. Humanistic-altruistic feelings and actions provide the basis for humanistic caring and promote professional care [3].

Caring ability in nursing refers to the ability to externalize humanistic literacy in clinical work [3]. It includes the ability to listen to patients' needs and desires, understand their emotions, communicate with them, feel the value of their life, and ultimately serve patients consciously and creatively [1, 3]. Individuals with high caring ability provide effective clinical practice and offer high quality care in hospitals, which contributes to important metrics such as high patient satisfaction, less work pressure, and more harmonious nurse-patient relationship [4]. Caring ability is an essential characteristic of a competent nurse [5], and more importantly, it is the core of nursing profession [6]. As an important backup force of clinical nursing work, nursing students’ caring ability will have a significant influence on the quality of clinical nursing in the future [7]. Thus, nursing students’ caring competence upon graduation from nursing programs must be guaranteed [1, 7].

Nursing students' caring ability may not be inherent but gradually developed through continuous experience and learning under the dual role of environment and education [1, 8]. Internship can be a vital part of both the environment and education. During internship, students spend a designated period of time in professional settings to practice what they learned in schools, and develop professional skills under the supervision of registered nurses [9, 10]. For nursing students, internships are the link between systematic education and clinical work [11]. Therefore, the internship period may affects the development of nursing students' caring ability [12].

Studies revealed contradicted findings regard the influence of internship on the caring ability of nursing students. One study found that the caring ability of nursing students with work experience is higher than that of students with no experience [13, 14]. Similarly, Ferri et al. [15] found that nursing students perceive a high level of caring ability before internship, and it significantly improve during internship. However, a descriptive study (2016) conducted in China compared the changes in undergraduate nursing students' caring ability before, during, and after internship showed an overall downtrend [8]. This was the only study conducted to explore caring ability of nursing students during internship in China. The study included 67 undergraduate nursing students from Northern China. The small, non-representative sample used might have limited the generalizability of the results. Furthermore, the study did not describe the plan or facilitation of internships. In addition, the study emphasized the personal influence of nursing students and teachers on caring ability while ignoring the possible impact of internship plan and facilitation process [8].

In China, no unified plans for nursing students’ internships are implemented. Internship programs are formulated independently by each nursing college. The difference in various aspects of internship among nursing schools such as education level, hospital level, clinical supervisors, and so on, have led to great variation in the facilitation of internship plans, resulting in difference in nursing students’ caring ability. This study was conducted to investigate nursing students’ self-perceived caring ability before and after internship in the south-central China. In this study, we sought answers to the following research questions: 1) What are undergraduate nursing students’ self-perception of caring ability? 2) Is there any differences between undergraduate nursing students' perception of caring ability before and after internship?

## Methods

### Study design

This was a descriptive longitudinal study. The data were collected from year 2018 to 2020.

### Setting and sample

Convenience sampling method was used to recruit undergraduate nursing students (*n* = 305) from one nursing school interned during 2018–2020. Sample size included 106, 124, and 75 students from 2018, 2019, and 2020 respectively. A questionnaire was used to collect data before and after internship. The inclusion criteria included nursing students who understood the purpose of study, agreed to participate, and had no prior internship experience. The exclusion criteria included nursing students who were unwilling to participate and those who had not completed the internship.

### Internship program design

The curriculum for undergraduate nursing students in participated nursing school consisted of two phases: Phase I, understanding basics of nursing and medicine (3 years), and Phase II, internship (1 year). Nursing students interned in their fourth year. The entire internship program was developed by the nursing school together with three internship hospitals. The program included preparation and internship sections (please see procedure of the internship program in the Appendix).

Preparation section had three phases: hospital assignment, pre-internship training, and mobilization meeting. In hospital assignment, students were randomly assigned to one of the three affiliated hospitals for one-year internship. All the three hospitals were tertiary general hospitals with similar departments and personnel structures. In pre-internship training, students participated in a two-week pre-internship training to strengthen their clinical nursing skills such as intravenous and intramuscular injection, cardiopulmonary resuscitation, and so on. Students were then required to pass skills evaluation before starting the internship. In mobilization meeting, details regarding aims, significance, regulation, and arrangement (e.g., assignment of interns to specific nursing units) of the internship were explained to the students.

Internship section included five phases: preceptors’ selection and training, unit introduction, preceptorship, post-preceptorship examination, monthly feedback and adjustment, mid-term nursing rounds, and summary meeting. In preceptors’ selection and training, registered nurses with a bachelor’s degree or above, having more than five years of working experience, good at communication and providing humanistic care were selected as preceptors. All preceptors received training for coaching students to practice nursing skills and to provide humanistic care for patients. Then, students started internship in selected nursing units (*n* = 12) for one month each. During unit introduction, preceptors introduced students with type of patients, medicines used, patients’ special nursing needs, and so on, before starting the internship at each unit.

Preceptorship is a teaching approach whereby students are individually assigned to staff nurses in the clinical practice setting [16]. Specifically, students were encouraged to communicate with patients when providing nursing care, for example, comforting patients when performing intrusive operations (e.g., urinary tube insertion), or providing pre-surgery education under the supervision of preceptors. The preceptorship aimed to develop basic nursing skills and caring ability of nursing students at the same time, promote the socialization of nursing students into the nursing profession, and the acquisition of professional values and identity. Students took a knowledge test and skill assessment after the internship at each unit. The knowledge test evaluated their knowledge of patient type in the unit, patients’ special nursing needs, and medicines used. Skill assessments evaluated students’ basic nursing skills and the ability to provide humanistic care in different scenarios. In the monthly feedback and adjustment stage, students gave feedback to the hospital nursing department regarding the one-month internship in the previous nursing unit. Six months after the internship started, the nursing school organized a mid-term assessment to evaluate students’ nursing skills and caring ability, by having students perform nursing rounds independently in front of nursing teachers and preceptors. Teachers and preceptors then provided feedback on nursing skills and patient care facilitation according to students’ performance. At the end of the 12-month internship, a summary meeting was conducted by the nursing school to receive feedback from students to the school and hospitals regarding the one-year internship, and the internship plan would be adjusted accordingly. During the meeting, students also shared their internship experience and suggestions for providing humanistic care with students newly started the internship.

### Measurement

Nursing students’ caring ability was measured before and after internship using the same questionnaire. The questionnaire consisted of two sections. The first section included sociodemographic characteristics such as age, gender, internship year, residence, and number of siblings.

The Caring Ability Inventory (CAI) developed by Nkongho [16] was used in the second section to measure the caring ability of participants. The English version of the CAI was translated into Chinese [17] and has three dimensions: cognitive (14 items), courage (13 items), and patience (10 items). The inventory has Cronbach’s alpha ranging from 0.67 to 0.80. Each item was scored in a scale of 1 (completely oppose) to 7 (fully agree). Thirteen items were reverse scored, with an overall score ranging between 37 to 259. The higher the score, stronger the caring ability. A score < 203.10 indicates low caring ability, a score between 203.10–220.30 indicates moderate level caring ability, and a score > 220.30 indicates high caring ability. In cognition, courage, and patience dimensions scores were at mid-level ranging 76.40 to 84.0, 62.50 to 74.0, and 61.0 to 65.2 respectively. Same as the overall score: a high level refers to a score exceeding the maximum value of medium level, and a low level refers to a score lower than the minimum value of medium level.

### Data collection

The pre-internship questionnaires were distributed to participants during motivation meeting. The research purpose was explained to the nursing students and those who agreed to participate were given an informed consent. The post-internship questionnaires were distributed at the summary meeting, and only those who consented and participated in the pre-internship survey were given the questionnaire. Questionnaires that were incomplete and had the same answer for all questions were identified as invalid questionnaires and excluded. From 2018 to 2020, 305 paired questionnaires (refers to questionnaires answered by the same student before and after internship) were sent out, and 300 valid paired questionnaires were collected.

### Ethics considerations

This study was approved by the institutional review board of participated nursing school (Approval No. E201896). The research participants were informed that their participation is voluntary and anonymous, they were also informed about the significance of research and required to sign informed consent before participation. No personal information of participants and participating hospitals were collected.

### Data analysis

Data were analyzed using Software Package Statistical Analysis (SPSS) Version 22.0 [18]. Descriptive statistics were used to analyze the demographic characteristics of the participants, and the Mean ± SD was used to present the CAI scores. For statistical inference, a paired t-test was used to compare the overall caring ability and three CAI dimensions of students before and after internship, considering statistical significance at a *p* value less than 0.05.

## Results

### Demographic characteristics

Valid paired questionnaires were collected from 300 students (response rate = 98.36%). Students had a median age of 21 (19–23) years, and around 90% (*n* = 268) of them were female; half of them came from urban areas (*n* = 174) and had sibling(s) (*n* = 151). The largest proportion of students interned in 2019 (*n* = 121), followed by 2018 (*n* = 104), and 2020 (*n* = 75). See details in Table 1.

**Table 1:** Demographic characteristics of students

Descriptive	Characteristics	
	***n***	***%***
**Gender**		
Male	32	10.67
Female	268	89.33
**Internship year**		
2018	104	34.67
2019	121	40.33
2020	75	25
**Student Leader**		
Yes	84	28
No	216	72
**Residence**		
Urban	174	58
Rural	126	42
**Has sibling(s) or not**		
Yes	151	50.33
No	149	49.67

### Comparison of nursing students' caring ability before and after internship

A comparison of the CAI scores of nursing students before and after internship is shown in Table 2. The overall CAI mean scores increased from 174.08 ± 17.72 before to 181.83 ± 16.28 after the internship, showed a significant improvement (*p* < 0.05). The cognitive and patience dimensions post-internship were also significantly improved (*p* < 0.05), while no significant change was found in the courage dimension.

**Table 2:** Comparison of CAI scores before and after internship (Mean ± SD)

Dimension	Before	After	t	p
Cognitive	71.05 ± 7.66	74.92 ± 8.62	5.44	.000
Courage	48.47 ± 12.46	49.75 ± 7.60	1.44	.152
Patience	54.11 ± 4.30	57.41 ± 5.12	7.95	.000
Total score	174.08 ± 17.72	181.83 ± 16.28	5.27	.000

*SD* standard deviation

## Discussion

This study explored the caring ability of undergraduate nursing students before and after internship. Our findings indicated an average low level of caring ability in nursing students [19]. This aligned with the results of a cross-sectional survey on caring ability of Chinese nursing students [20]. The low level of caring ability might be due to the motivation of nursing students to enter nursing programs and the focus of caring ability courses. According to Cai et al. [21], only 35% of Chinese students entering nursing programs aspire to become nurses. Majority of the students studied nursing to get good job opportunities or because of being transferred from first-choice programs [22]. Thus, these students were more likely to be passive in performing patient care. In addition, nursing care in China has long been a disease-centered model and was transferred into patient-centered care only since the last 20 years [23]. The undergraduate nursing education in China focused more on improving nursing professional knowledge and clinical nursing skills of students [20]. In 2011, The Chinese Nursing Development Plan (2011–2015) [24] recommended that nursing schools increase the humanities content in their curriculum to strengthen caring consciousness. Since then, nursing schools begun to offer caring ability courses after recognizing its importance in facilitating patient-centered care [5]. However, these courses are still in their infancy [5]. The caring ability courses in Chinese nursing schools only account for 8% of the total courses, far less than 15–25% in countries such as the US, UK and Germany [25]. In addition, most courses only focus on theoretical education and fail to offer clinical training opportunities [26]. Nurses' caring ability is determined by how accurately they identify patient needs and perform appropriate caring behaviors through interactions with patients [27]. Caring ability courses should provide more opportunities for students to rehearse interpersonal skills in the classroom environment, and interaction with patients at the clinical settings [28]. More studies are needed to explore efficient ways to nurture the caring ability of nursing students in China.

Our findings revealed that the overall caring ability of nursing students significantly improved after internship. This can be attributed to the implementation of the internship program, which emphasized on cultivating caring ability while developing students’ basic nursing skills. Notably, students were interned under the preceptorship of experienced clinical nurses with high level of caring ability [29]. During the preceptorship, students learned the ways to build relationship and interact with patients by observing and imitating preceptors’ nursing practice [30]. They also learned to recognize patients’ experiences, attended to patients’ perspectives, and thereby realized their own professional obligations to advocate for, and provide comprehensive and holistic care to patients [31]. This finding aligned with Yu et al.’s [26] study, which suggested that sustained support and guidance were critical for students to perform and develop caring ability. Vihos [31] et al. also claimed that preceptors are instrumental in creating safe spaces for nursing students to interact with patients, explore encounters and moral issues in practice, and build confidence in caring for patients therefrom. Moreover, students’ caring ability was further enhanced by monthly evaluations, mid-term nursing rounds, and feedback and adjustment.

The cognitive scores ranked the highest amongst the three CAI dimensions, followed by patience, and courage, which is consistent with the findings of Chen et al. [19]. This might be because nursing is a female-dominated profession; female nursing students accounted for 90% of all participants in our study. They exhibit more patience but less courage to actively communicate with patients, especially for nurse interns with less caring experience [19, 32]. Another alerting point concerns the current caring ability courses in China. Several studies warranted that these courses emphasized more on improving students’ understand of the significance of caring in the nursing profession, and cultivating their patience in patient care. While ignoring the importance of developing the courage to actively interact with patients [33, 34]. Moreover, the self-perceived cognitive and patience levels of students improved significantly with the supervision and encouragement of preceptors, while no significant change was found in their courage. Similarly, Wu et al. [33] found that even though preceptorship was among the most effective ways to improve the caring ability of nurses, the least effect was found on courage dimension. Our findings pointed out that it is difficult to implement courage in the caring process since it requires more experience and sensitivity toward patient care [33]. Studies revealed that the courage dimension had a close relationship with years of clinical nursing, senior nurses had higher scores on courage than new nurses [33]. It is understandable since senior nurses with more clinical nursing experiences possessed a higher level of nursing skills and confidence in communicating with patients, and thereby had more courage to do so. Our study indicated that one-year internship was too short for nursing students to show significant improvement in the courage dimension. It also suggested that nursing schools should provide students with more opportunities to expose to the clinical environment and interact with patients to gain more clinical experience and improve their confidence and courage to provide patient care [34].

This is the second longitudinal study conducted in China to evaluate the caring ability of undergraduate nursing students before and after internship. A previous study conducted in Northern China showed a downtrend in caring ability during internship [8]. The difference in the internship programs might have contributed to the distinct results. Internship is a critical period for nursing students. Therefore, a well-developed internship program must be implemented to improve the nursing students’ caring ability.

## Limitations

This study has several limitations. The findings obtained were based on nursing students’ self-evaluation. To acquire a comprehensive assessment of students’ caring ability level, future studies could assess objective perceptions of preceptors and patients. In addition, the sample does not represent overall nursing education programs in China since our study was conducted in only one university in the Southern Central area of China. While the description of the internship content development, implementation and evaluation might provide reference to future internship planning for undergraduate nursing students in China or other countries.

## Conclusions

Our findings revealed that the caring ability of undergraduate nursing students in China were at a low level. It also showed that the overall caring ability of nursing students significantly improved after internship, indicating a positive relationship between internship and caring ability. The courage level of nursing students remained low before the internship and showed no-significant improvement afterwards. Future research is needed to explore the effectiveness of internship in developing caring ability, especially the courage dimension, and to identify relevant factors influencing the process. We suggest that nursing educators and clinical nurses emphasize the importance of caring ability development in internship planning, and encourage nursing students to engage more with patients.

## Supplementary information

**Additional file1:** **Appendix**. Internship program for undergraduate nursing students.

## Data Availability

The datasets generated and/or analysed during the current study are not publicly available due to limitations of ethical approval involving the anonymity but are available from the corresponding author on reasonable request.
