# Supplementary material for: Caring ability of nursing students pre- and post-internship: a longitudinal study
Source: BMC Nurs. 2022 May 30;21:133. doi: 10.1186/s12912-022-00921-2 (PMC9150307; doi:10.1186/s12912-022-00921-2)
Supplement: Supplementary file 1 — Additional file1: Appendix. Internship program for undergraduate nursing students. [file 12912_2022_921_MOESM1_ESM.docx]

**Appendix. Internship program for undergraduate nursing students**

**
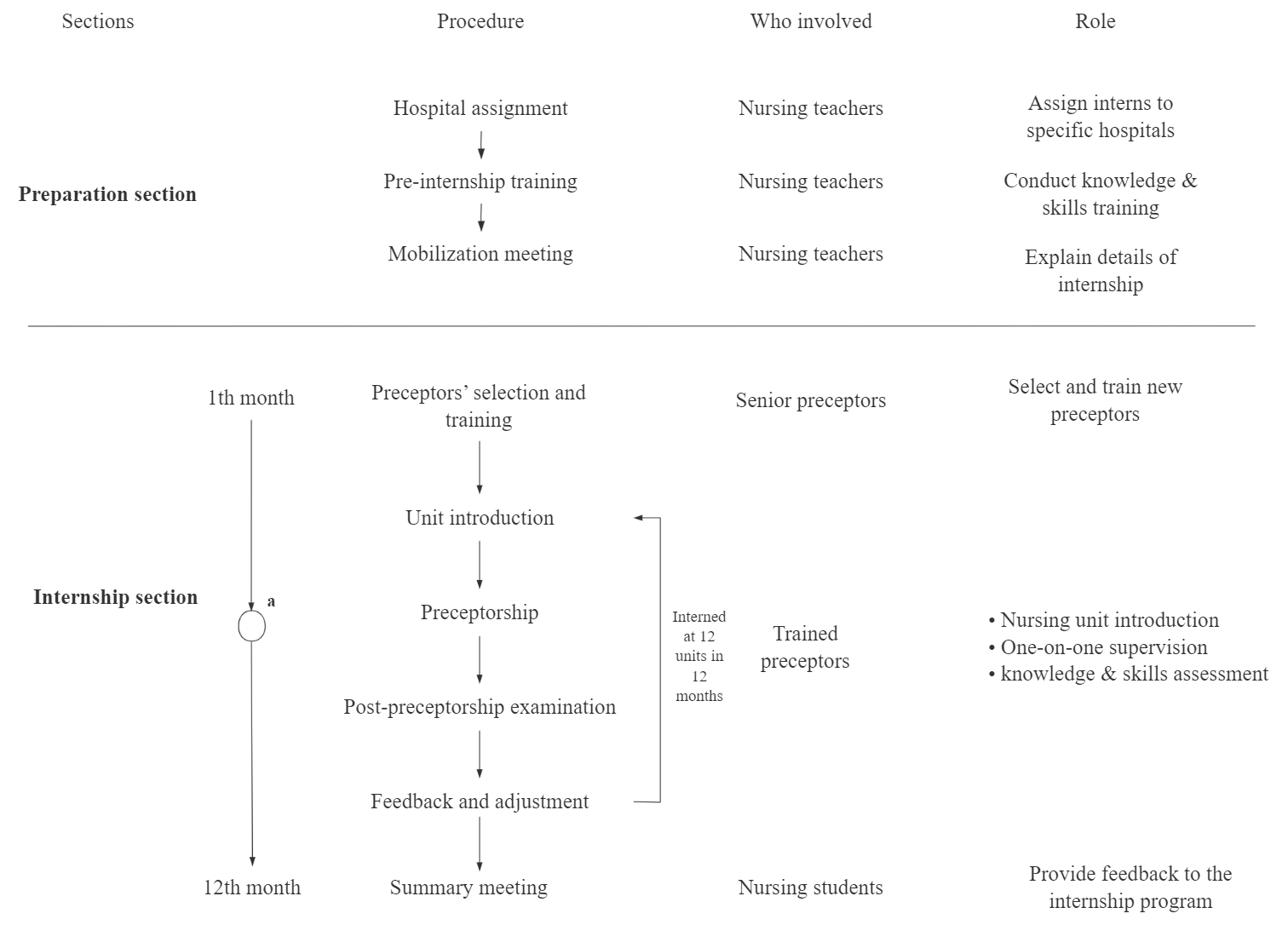
**

**^a^** There are mid-term nursing rounds at six months after the internship begins, nursing teachers and preceptors evaluate and provide feedback based on students’ performance in the nursing rounds.
